# Cumulative Risk, Cumulative Outcome: A 20-Year Longitudinal Study

**DOI:** 10.1371/journal.pone.0127650

**Published:** 2015-06-01

**Authors:** Leslie Atkinson, Joseph Beitchman, Andrea Gonzalez, Arlene Young, Beth Wilson, Michael Escobar, Vivienne Chisholm, Elizabeth Brownlie, Jennifer E. Khoury, Jaclyn Ludmer, Vanessa Villani

**Affiliations:** 1 Ryerson University, Toronto, Ontario, Canada; 2 Centre for Addiction and Mental Health, Toronto, Ontario, Canada; 3 University of Toronto, Toronto, Ontario, Canada; 4 McMaster University, Hamilton, Ontario, Canada; 5 University of Guelph, Guelph, Ontario, Canada; 6 Social Planning Toronto, Toronto, Ontario, Canada; 7 Queen Margaret University, Edinburgh, United Kingdom; University of New South Wales, AUSTRALIA

## Abstract

Cumulative risk (CR) models provide some of the most robust findings in the developmental literature, predicting numerous and varied outcomes. Typically, however, these outcomes are predicted one at a time, across different samples, using concurrent designs, longitudinal designs of short duration, or retrospective designs. We predicted that a single CR index, applied within a single sample, would prospectively predict diverse outcomes, i.e., depression, intelligence, school dropout, arrest, smoking, and physical disease from childhood to adulthood. Further, we predicted that number of risk factors would predict number of adverse outcomes (cumulative outcome; CO). We also predicted that early CR (assessed at age 5/6) explains variance in CO above and beyond that explained by subsequent risk (assessed at ages 12/13 and 19/20). The sample consisted of 284 individuals, 48% of whom were diagnosed with a speech/language disorder. Cumulative risk, assessed at 5/6-, 12/13-, and 19/20-years-old, predicted aforementioned outcomes at age 25/26 in every instance. Furthermore, number of risk factors was positively associated with number of negative outcomes. Finally, early risk accounted for variance beyond that explained by later risk in the prediction of CO. We discuss these findings in terms of five criteria posed by these data, positing a “mediated net of adversity” model, suggesting that CR may increase some central integrative factor, simultaneously augmenting risk across cognitive, quality of life, psychiatric and physical health outcomes.

## Introduction

Cumulative risk (CR) models involve identifying a set of proven risk factors (e.g., low maternal education, maternal depression, father absent), dichotomizing them (as extant or not), and tallying them to derive a risk score of 0 (no risk factors) to some upper limit (representing all risk factors considered) for each individual in a given sample. Multiple risks are thereby combined into a single index to predict an outcome of interest. Rutter’s [1] seminal work illustrates the CR approach. Rutter identified six environmental risk factors associated with child psychiatric disorder. He then constructed a “family adversity index,” assigning each child a score of 0 to 4+, reflecting number of identified risk factors present in the child’s life. Rutter found a striking association between number of risk factors and probability of psychiatric disorder: Children with 0 or 1 environmental risk factors showed about 2% probability of being diagnosed with a disorder. However, this probability increased with every risk factor, such that children with 4+ risk factors showed over 20% likelihood of diagnosis. This pattern held no matter what single stressors were involved, i.e., *it was not the particular stressor that rendered the child vulnerable to psychopathology*, *but the number of stressors*, an oft-replicated finding [2–7]

CR predicts an extraordinary range of outcomes. For example, using similar CR indices, researchers have shown negative impact across motor development [8], intellectual performance [6,7], academic standing [5], self-esteem [9], extracurricular participation [9], executive function [10], emotion regulation [11], psychiatric status [1], physiological responsivity [11,12], tobacco use [13], and physical health [14]. The fact that similar CR indices are linked to diverse outcomes across varied samples illustrates the broad impact of CR. In fact, Evans [11] showed that a single CR index was related to delayed gratification, learned helplessness, internalizing and externalizing behaviours, heightened cardiovascular and neuroendocrine parameters, and increased deposition of body fat *in a single sample* of low income families. Such findings are important because most studies of predictor-outcome relations focus on a single predictor [4,15]. Even more so, they focus on a single outcome, disregarding the probable co-occurrence of adversity. The breadth of empirical findings suggests that CR may offer profound insights into coordinated developmental trajectories that incorporate a host of interrelated outcomes.

Despite the exceptional power and consistency of CR findings, the theoretical impact of CR models is blunted by several factors. 1) The research remains largely unintegrated (c.f., [4]). For example, researchers in the areas of academic function and physical health typically do not cross-reference one another, although they may use comparable CR indices. Furthermore, with few exceptions, investigators assess one outcome at a time. When they assess more, all outcomes usually fall within a single domain (e.g., mental health but not physical health; c.f., [11]). 2) Most CR findings derive from cross-sectional studies. Prospective longitudinal work is rare, particularly as it involves multiple assessments over time, and rarely encompasses early childhood to adulthood. This leaves us blind to temporal parameters, a potentially important oversight [4]. For example, Appleyard et al.[2] followed a cohort from birth to age 16 and assessed CR at multiple time points. They found that middle childhood CR did not predict adolescent emotional/behavioral problems, while early childhood CR did, and that early CR significantly predicted both internalizing and externalizing problems even after partialling out the effect of middle childhood CR. These results suggest a special sensitivity to adversity in early childhood (see also [16,17]). Such findings have important theoretical implications for the CR paradigm and for understanding the integrated nature of development more generally. 3) CR research is a purely empirical endeavour; little theory precipitated the approach or emerged from it, although such work is necessary to understand findings [4]. We address these issues here, predicting from CR in childhood and adolescence to diverse outcomes in adulthood.

In their review of the CR literature, Evans and Whipple [4] recommended the inclusion of multiple risk domains in CR studies. They noted that the most basic demarcation amongst domains involves the distinction between environmental and individual risk factors. Inclusion of these domains enables assessment of their relative influence and their interactive effects. In this regard, S/L impairment represents an important aspect of the child him/herself (in contrast to the purely environmental factors mentioned above), because it is so integrally involved in negotiating the interpersonal environment. Like CR, S/L impairment is related to an array of adverse outcomes, including continued language and communication difficulties, impaired social skills, poor academic performance, cognitive deficits, internalizing and externalizing behavior problems, increased use of substances, and psychiatric illness [18,19]. Given its broad impact, Snow and Powell [20] argued that language disorder is a public health issue that must be conceptualized within a broader risk framework. With these considerations in mind, we incorporated S/L impairment as a potentially powerful covariate and moderator of the impact of CR on adverse outcome [21].

Based on the above survey, we hypothesized as follows. (1) A single CR index, assessed in childhood and adolescence, is related to multiple adverse outcomes, i.e., depression, intelligence, smoking, high school dropout, arrest, and physical disease, as assessed in adulthood. (2) By extension, CR is related to cumulative outcome (CO), i.e., the more risk factors experienced, the greater the number of adverse outcomes. (3) Early CR explains variance in CO, the major dependent variable in this study, over and above that attributable to later CR. In addition, (4) we assessed the impact of cumulative environmental risk over and above a potent individual predictor, child speech/language disorder. (5) We explain the seemingly complex findings with a parsimonious model pertaining to CR and integrated development.

## Method

Mothers provided written informed consent on behalf of their children at 5 and 12 years old. Children gave written consent at age 12 and each subsequent age. All stages of this research were conducted following approval from the Royal Ottawa Hospital or Centre for Addiction and Mental Health Research Ethics Boards, as appropriate.

### Samples

We used two samples, one consisting of children with speech/language (S/L) impairments, the other of matched peers without S/L impairment. The samples were combined and S/L impairment was covaried in analyses where necessary. Participants were assessed at ages 5/6, 12/13, 19/20, and 25/26 years.

Screening was initiated involving a one-in-three random sample of all 5-year-old English-speaking children in the Ottawa-Carlton region of Ontario, Canada [22]. Of the 1,655 children selected, 94% agreed to screening for speech and language impairments (described below). In all, 142 children identified with speech and/or language impairments agreed to continued participation. A control sample of 142 children matched on age and sex and from the same classroom or school was selected from those who passed the initial screening, as detailed elsewhere [22,23]). Ethnicity was not formally assessed but we estimate that the sample was between 90 and 95% White, reflecting local demographics at the onset of this study [24].

In 1989, when the children were 12/13 years old, 244 subjects (89% of original sample) agreed to re-assessment [25]. In 1996, when participants were 19/20, 258 (90.8% of the original sample) were re-assessed [19]. In 2002–2003, when participants were 25/26, 244 (86% of the original sample; 112 from the S/L group, 132 from the control group) were re-assessed. As in earlier waves, the 25/26-year assessment battery included language measures, cognitive and academic tests, psychiatric measures, and demographic information (described below). The protocol took about 5½ hours to complete. Subjects were paid $100.00 CDN to defray participation costs. Complete data were available for 184 participants on the 31 variables used in this study across 20 years. As described below, multiple imputations were conducted to account for missing data. The resulting sample size was 284; 137 individuals diagnosed with S/L impairments, 147 controls. The sample included 183 (64.43%) males.

### Measures

#### Speech/language

We screened participants with language and speech tests at age 5/6 using the Bankson Language Screening Test [26], Screening Test for Auditory Comprehension of Language [27], Photo Articulation Test [28], and a screening for voice disorders and stuttering (see [22]). At all ages language skills were further assessed with the Peabody Picture Vocabulary Test-Revised (PPVT-R, [29]), the age-appropriate version of the Tests of Language Development (TOLD, [30]; TOLD-Intermediate, [31]; TOAL-3, [32]), and the Goldman-Fristoe-Woodcock Auditory Memory Tests ([33]; at Time 1 and Time 2). Participants were diagnosed with language impairment if they scored one standard deviation below the mean of the published norms on any full test or two standard deviations below the mean of any TOLD/TOAL-3 subtest (we used published local norms for the TOAL-3 [34]).

Checklists completed by a speech-language pathologist concerning the presence of articulation, voice, and fluency problems were used to determine speech impairment. Children diagnosed with a voice disorder, stuttering or dysarthria, or who scored two standard deviations below the mean on the Word Articulation or Word Discrimination subtests of the TOLD, were classified with speech impairment [35,36].

#### Risk and outcome

At ages 5/6, 12/13, and 19/20, we collected data on socio-economic status, maternal age at birth of first child, family size, maternal depression, parental marital status, and parental criminal conviction. A CR score was tallied based on status on each item. For one item, maternal age at birth of first child, status could not change over time. The use of unvarying risk factors in longitudinal CR designs is not new [6]. In the case of parental criminal conviction, stability was artifactually magnified because once a parent had been convicted his/her status cannot change.

At age 25/26, we constructed a 6-item “cumulative outcome” index. The index consisted of: depression, FSIQ, smoking, school dropout, arrest, and physical illness. Using the CR index, we attempted to predict each outcome separately as well as the total number of outcomes (CO). We derived risk and outcome factors as follows.

### Cumulative risk index

1. *Socio-economic status* (SES) was determined according to father's or mother's occupation, whichever was higher, using a Canadian occupations index [37]. This index has a mean of 42.74 (standard deviation (SD) = 13.28). The means of the current combined sample were 47.29, 51.81, and 50.12 (SD = 15.16, 14.41, 14.56) at 5/6, 12/13, and 19/20 years, respectively. Families in the lowest quartile were assigned to the risk category.
2. *Maternal youth at birth of first child* was considered a risk factor, with children of mothers whom had given birth before age 20 categorized as at risk. Age 20 cut-off defines adolescent pregnancy and is recognized by demographers as a risk factor [38,39].
3. *Family size* was considered a risk factor when four or more children were in the household. This criterion has varied in the CR literature, with 3 [40], 4 [41], or 5 [42] or more children considered a risk factor. Canadian family size statistics for 1981, when this study was undertaken, indicate that 4.9% of parents had 4 or more children [43].
4. *Maternal depression* was assessed with the Center for Epidemiologic Studies Depression Scale (CES-D [44]) at each time point. The CES-D has high internal consistency and retest reliability. CES-D scores correlate highly with other self-report and clinical ratings of depression [44]. Mothers were assigned to the risk category if their scores were higher than 15, as recommended in the manual [44].
5. *Maternal marital situation* was considered a risk factor if the child lived in a single-parent home or if mother reported low marital satisfaction. The combination of single-parenthood and high marital dissatisfaction is commonly used in CR research (e.g., [1,42,45]); both represent disrupted parental relations. With respect to single parenthood, it has been shown in a large Canadian sample that single mothers, as compared to married mothers, were more likely to report depression, chronic stress, negative life events, and low social support [46]. Marital satisfaction was assessed at each time point with the Short Marital Adjustment Test [47]. It has high internal reliability and discriminates between couples seeking clinical service for marital problems and nonclinic couples [48]. Locke and Wallace [47] proposed that scores less than 100 indicate clinical levels of distress and we used this cut-off to indicate risk status.
6. Self-reported *parental conviction* (either parent) for at least one criminal offence was considered a risk factor. The use of self-reported parental criminal conviction is commonly used in the CR literature [49–51].

### Cumulative outcome index

1. Depression was assessed with the CES-D, clinical cut-off 15, as described above.
2. Intellectual functioning was assessed with a four-subtest (Vocabulary, Similarities, Picture Completion, Block Design) combination short form of the Wechsler Adult Intelligence Scale-Third Edition (WAIS-III [52]). Applying Tellegen and Briggs’ [53] formulae (and Atkinson and Yoshida’s, [54] software) to Canadian norms, the reliability and validity of the 4-subtest short form are r = .92 and .90, respectively. For the purposes of the CO index, we considered an adverse outcome to involve a FSIQ more than 1 SD below the normative mean (< 85; FSIQs of 85 to 115 are considered “average”[52]).
3. We asked participants whether they had completed high school and, if not, whether they had subsequent training. We considered incomplete high school with no further training an adverse outcome.
4. We asked participants how many cigarettes they smoked per day, ranging from 0 to 20+. Although the validity of self-reports is confounded by features like type of cigarette and exposure to environmental tobacco smoke [55], self-reports are reliable [56] and valid [57,58] when assessed against biochemical assays in national surveys. At least one cigarette/day was considered an adverse outcome; such frequency is a significant health hazard [59].
5. We asked participants whether they had been arrested since age 19/20, with an affirmative response considered adverse. There is debate regarding the relative merits of self-reported criminal involvement versus official arrest records, but concordance is generally high [60], with a low rate of under-reporting [61].
6. We asked participants whether they had been diagnosed with any of the following over the past 12 months: ear infection, allergies, asthma, cancer, epilepsy, arthritis, heart problems, muscle disease, diabetes, kidney disease, or convulsions with a fever. These diseases are either chronic (diabetes, asthma), represent major organ systems (such as the heart and the kidneys), or are referable to the nervous system (such as epilepsy). The issue of ear infections is related to concerns about hearing and S/L development. We considered affirmation of at least two illnesses an adverse outcome.

### Analyses

#### Multiple imputation

Multiple imputations were conducted to account for missing data with respect to risk and outcome factors. Multiple imputation addresses missing data by replacing it with *x* > 1 sets of simulated imputed cells, resulting in *x* plausible but unique versions of the complete dataset. Each of the *x* datasets is analyzed uniformly and is then combined to yield overall estimates and standard errors that reflect sample variation and missing-data uncertainty [62]. Twenty imputations were conducted for the current analyses, exceeding recommended minimum and sufficient to obtain adequate inferences [62–64]. The average of the 20 imputations for each model’s significance and the pooled predictors were utilized. To determine if the data were suitable for imputation, Little’s [65] missing completely at random (MCAR) test was conducted. MCAR revealed that the data for analyses with age 5/6 CR, χ^2^ (2) = 1.81, *ns*, age 12/13 CR, χ^2^ (2) = 1.36, *ns*, and age 19/20 CR, χ^2^ (2) = 5.80, *ns*, were missing at random, and thus imputation was appropriate [62].

## Results

### Background Analyses

We conducted several background analyses to characterize the samples, evaluate the prevalence of risk and adverse outcome, and assess the intercorrelations amongst risk and outcome variables.

### Risk Factors

The number of families scoring positively on any given risk factor varied from 36 (12.8%; > 4 children, age 5/6) to 136 (48.0%; single parent or dissatisfied in marital relationship, age 12/13). Families with S/L impairment showed a significantly greater probability of disadvantage compared to families without S/L impairment in the areas of low SES (*X* ^2^ (1) = 6.05, *p* < .05) and maternal age at birth of first child (*X* ^2^ (1) = 5.10, *p* < .05); group differences were nonsignificant for parent conviction (*X* ^2^ (1) = 1.72, *p* = .19), > 4 children (*X* ^2^ (1) = 2.79, *p* = .09), marital status (*X* ^2^ (1) = 1.81, *p* = .18), and maternal depression (*X* ^2^ (1) = 1.36, *p* = .24). The average number of environmental risk factors at Times 1, 2, and 3 varies from 1.23 to 1.58, indicating that this is a low-risk sample. This presents a stringent test of hypotheses. Nevertheless, study of low-risk samples is important in the contexts of typical development and developmental psychopathology, both of which seek to explain variability under conditions of low risk [66] and common principles in normal and abnormal development [67].

Mann-Whitney U tests revealed significant differences between the S/L and control samples with respect to CR at all ages; age 5/6 (*U* = 8146.42, *p* < .01), age 12/13 (*U* = 6708.28, *p* < .001), age 19/20 (*U* = 7174.93, *p* < .001), with the S/L sample showing more risk than the control sample in every case. In terms of CR stability, Spearman *rho* correlations of .73 (Time 1 to Time 2), .61 (Time 1 to Time 3), and .81 (Time 2 to Time 3; p < .001 in every case), indicated substantial and significant stability or chronicity over time. (When those items with restricted potential variation (i.e., maternal age at first child, history of parental conviction) are both removed, stability remains high; .60 from Time 1 to Time 2, .44 from Time 1 to Time 3, .68 from Time 3 (p < .001 in every case)).

We assessed relations amongst risk measures using the highest metric possible in each case (Table 1, off-diagonal entries). Several partial correlations, controlling for S/L impairment, were significant. Overall, the data show interrelatedness amongst risk factors, typical of CR indices [4].

**Table 1:** Partial Intercorrelations and Stability Coefficients among Risk Factors.

Risk Factors	1	2	3	4	5	6
1. Parent SES	.53^#^/.35^#^/.61^#^					
2. Maternal age, birth	.28^#^/.18^+^/.20^+^	___				
3. Number sibs in home	.07/.07/.13*	.05/.01/.21^#^	.67^#^/.30^#^/.36^#^			
4. Maternal depression	.17^+^/.30^#^/.10	.14*/.06/-.04	.16^+^/-.08/.08	.30^#^/.28^#^/.43^#^		
5. Marital situation	.27^#^/.23^#^/.27^#^	.10/.13*/.04	.06/-.05/.03	.26^#^/.20^+^/.20^+^	.44^#^/.35^#^/.51^#^	
6. Parent conviction	.28^#^/.37^#^/.21^#^	.24^#^/.37^#^/.32^#^	-.02/.03/.17^+^	.05/.22^#^/.03	.31^#^/.26^#^/.08	.73^#^/.86^#^/.77^#^

Note. Partial correlations among risk factors, controlling for S/L status; Time 1/Time 2/Time 3; stability coefficients of risk factors in diagonal (Correlations of Time 1 with Time 2/ Time 1 with Time 3/Time 2 with Time 3); Maternal age, birth = maternal age at birth of first child;

*p < .05,

^+^p < .01,

^#^ *p* < .001.

### Outcome factors

The number of individuals with each outcome factor varied between 17 (6.0%; low FSIQ) and 142 (50.0%; smoking). Compared to the sample without S/L impairment, the sample with impairment included a greater percentage of individuals with any given outcome, however these difference were not significant for any outcome.

We assessed relations amongst outcome measures, using the highest metric possible in each case. Partial correlations, controlling for S/L impairment, revealed several significant correlations (see Table 2). Several correlations between individual risk and individual outcome variables also emerged as significant (Table 3).

**Table 2:** Partial Intercorrelations among Outcome Factor.

Risk Factors	1	2	3	4	5
1. Depression	-				
2. FSIQ	.16^+^	-			
3. Dropout	.14*	.16^+^	-		
4. Smoke	.13*	.02	.21^#^	-	
5. Arrested	.10	.08	.25^#^	.17^+^	-
6. Illness	.17^+^	.03	.10	.17^+^	.06

Note. Partial correlations among outcome factors, controlling for S/L status.

*p < .05,

^+^p < .01,

^#^ *p* < .001.

FSIQ = Full Scale IQ.

Dropout = incomplete high school.

**Table 3:** Partial Intercorrelations between Risk and Outcome Factors.

Risk Factor	Outcome					
	Depression	IQ	Incomplete school	Smoke	Arrested	Physical Disease
Parent SES	.21^#^/.11/.16^+^	.26^#^/.36^#^/.30^#^	.25^#^/.26^#^/.07	.05/.084/.07	.17^+^/.21^#^/.17^+^	.07/.09/.10
Maternal age, birth	.06	.02	.27^#^	.10	.30^#^	.09
Family size	.02/.06/.01	.05/.14*/-.01	.06/.00/.13*	.01/.04/.04	.04/-.01/.05	-.05/.04/.09
Maternal depression	.02/.00/.00	.12/.06/.10	.16^+^/.23^#^/.18^+^	-.04/.08/-.02	.08/.03/.07	.06/.11/.14*
Marital situation	.13*/.10/.08	.03/-.06/.06	.25^#^/.07/.01	.12/.16^+^/-.03	.18^+^/.17^+^/.05	.20^+^/.08/-.02
Parent conviction	.10/.08/.08	.08/.14*/.08	.18^+^/.26^#^/.17^+^	.14*/.09/.12	.17^+^/.25^#^/.18^+^	.19^+^/.20^+^/.17^+^

Note: Partial correlations controlling for S/L status; Time 1/Time 2/Time 3; Maternal age, birth = maternal age at birth of first child;

*p < .05,

^+^ *p* < .01,

^#^ *p* < .001.

We examined sex against each outcome to assess for potential confounding; for example, previous literature has shown that sex is linked to depression [68] and arrest [69]. We found that significantly more males than females reported a history of arrest (χ^2^ = 8.09, p < .01); otherwise, no significant differences emerged. We therefore included sex as a main effect in regression analyses involving arrest.

### Focal Analyses

#### CR and individual outcomes

We assessed relations between CR, on the one hand, and individual outcome variables, on the other. We combined S/L and control samples and conducted multiple regression (in the case of depression and FSIQ), binary logistic regression (in the case of incomplete high school, arrest, and physical illness) and Poisson regression (in the case of cigarettes per day and CO); in all instances, S/L status served as covariate. We conducted diagnostics for each analysis [70,71]. Assumptions were met, although we excluded two cases because Cook’s distance suggested their potentially undue influence. In the first round of regression analyses, we force entered S/L diagnosis, CR, and the diagnosis x CR interaction (plus sex in analyses involving arrest history). In no analysis did the interaction prove significant. To simplify analyses and sensitize them to main effects, we removed the interaction term from all equations and re-analysed the data. Results are shown in Table 4.

**Table 4:** Regression Results: Outcomes Regressed on S/L Impairment and Cumulative Risk at Each Age.

	Multiple Regression Results											
	Age 5/6				Age 12/13				Age 19/20			
	*Beta*	*SE*	*t*	*R* ^*2*^	*Beta*	*SE*	*t*	*R* ^*2*^	*Beta*	*SE*	*t*	*R* ^*2*^
Depression												
S/L impairment	2.08	1.56	1.80	.08	1.82	1.22	1.49	.07	1.85	1.20	1.55	.06
Cumulative risk	1.62	.43	3.77*		1.27	.47	2.67^+^		1.36	0.44	3.09^+^	
FSIQ												
S/L impairment	-10.72	1.76	-3.27^+^	.22	-10.44	1.81	-5.78^+^	.22	-10.43	1.81	-5.76^+^	.24
Cumulative risk	-3.17	0.69	-4.62^+^		-2.86	.69	-4.13^+^		-2.82	.77	-3.68^+^	
	Logistic Regression Results											
	Age 5/6				Age 12/13				Age 19/20			
	*Beta*	*SE*	*Wald*	*OR*	*Beta*	*SE*	*Wald*	*OR*	*Beta*	*SE*	*Wald*	*OR*
School Dropout												
S/L impairment	.85	.50	4.58	2.35	.52	.43	1.94	1.69	.69	.43	3.40	2.00
Cumulative risk	.75	.15	29.74^+^	2.13	.65	.17	26.64^+^	1.91	.55	.15	18.46^+^	1.73
History of Arrest												
Sex	1.61	.55	9.99^+^	5.02	1.86	.57	13.33^+^	6.44	1.79	.58	11.92^+^	5.99
S/L impairment	.082	.45	0.40	1.09	-.12	.46	.42	.89	-.31	.44	0.83	.73
Cumulative risk	.59	.14	20.75^+^	1.81	.69	.17	27.71^+^	2.00	.67	.17	24.14^+^	1.96
Physical Illness												
S/L impairment	.38	.29	2.44	1.47	.25	3.11	1.19	1.28	.35	.29	1.94	1.42
Cumulative risk	.28	.11	8.61*	1.32	.29	.11	10.57^+^	1.33	.30	.11	10.37^+^	1.36
	Poisson Regression Results											
	Age 5/6				Age 12/13				Age 19/20			
	*Beta*	*SE*	*Wald*	*pR* ^*2*^	*Beta*	*SE*	*Wald*	*pR* ^*2*^	*Beta*	*SE*	*Wald*	*pR* ^*2*^
Cigarettes per day												
S/L impairment	.02	.09	.00	.02	.02	.08	.03	.15	-.05	.08	.32	.02
Cumulative risk	.11	.03	13.29*		.13	.03	21.02^#^		.07	.03	5.53*	.11
Cumulative outcome												
S/L impairment	.23	.13	2.38	.11	.29	.15	4.00*	.09	.26	.15	3.07	.07
Cumulative risk	.20	.05	21.97^#^		.20	.05	18.53^#^		.18	.05	13.02^#^	

Note: *R* ^*2*^ refers to variance explained by complete model; *Wald* = Wald χ^2^ statistic; OR = odds ratio; S/L impairment = speech/language impairment as assessed at age 5/6 years; p*R* ^*2*^ = pseudo-*R* ^2^ as calculated with Coxe, West, & Aiken’s (2009) formula 9. The p*R* ^*2*^ represents how much closer the model is accounting for all the variance as variables are added. (It is not comparable to the *R* ^2^ derived from ordinary least squares regression, which represents proportion of variance explained.) We derived pseudo-*R* ^2^ by comparing the complete model to a model where no predictor variables were entered.

* p < .05;

^+^ p < .01;

^#^ p < .005

At all ages (5/6, 12/13, 19/20), CR significantly predicted every outcome—depression, FSIQ, high school dropout, smoking, arrest, and physical disease; the greater the CR, the greater or more likely the adverse outcome. In the case of FSIQ, the main effect of S/L impairment was also significant: Individuals with S/L impairment earned lower FSIQs than others. With respect to history of arrest, sex was also a significant predictor, with more males having such a history than females. Overall, the most consistent findings involved the significant main effect of CR on outcome (Table 4).

By way of caveat, we computed a Poisson regression in predicting number of cigarettes/day. We were unable to employ multiple imputation here for several reasons. Poisson regression is designed for count (i.e., integer) data, but multiple imputation generates noninteger values. Count data also have a lower limit of 0 and the pseudo-*R* ^2^ that one can generate in the context of Poisson regression represents deviation from 0; however, multiple imputation generates negative values. Therefore, in exploratory vein, we conducted multiple imputation but set 0 as the lower bound and rounded all decimals to the nearest integer. Using these data, however, we were unable to generate solutions that met all convergence criteria. Hence, we conducted the Poisson regressions using only subjects with complete data across the 31 variables and 20 years incorporated in this study (*N* = 184 minus the two outliers mentioned above). Compared to individuals with complete data, participants with incomplete data were more likely to have been diagnosed with S/L impairment (χ^2^ = 9.63, *p* = .002), to have dropped out of school (χ^2^ = 7.37, *p* < .01), and to earn a lower mean FSIQ (*t* = 3.24, *p* = .001). There were no differences respecting sex of participant, depression, arrest, cigarettes/day, physical illness, or CR at any age. Overall, it appears that more challenged individuals provided less complete data, as in other longitudinal CR studies (e.g., [72]). This likely restricts range and, together with the reduced sample size, may render the data less sensitive to differential outcome. Nevertheless, as mentioned above and shown in Table 4, at every age CR significantly predicted number of cigarettes/day.

#### Cumulative risk, cumulative outcome

To assess relations between CR and CO, we computed a Poisson regression; we regressed CO on S/L impairment and CR. Table 4 shows that CR significantly predicted CO at every age; the more risk factors, the more adverse outcomes. At age 12/13, there was also a main effect for S/L impairment. Again, Poisson regression was used to predict CO and all issues discussed immediately above pertained here.

#### Relations between earlier and later cumulative risk and cumulative outcome

Before examining whether age 5/6 CR improves model fit beyond age 12/13 CR and age 19/20 CR, we assessed for multicollinearity. We found no evidence of it (tolerance exceeded .20 for all independent variables and no condition index approached 15 [71]). Therefore, to assess the independent impact of age 5/6 CR on CO, we simultaneously entered S/L impairment and age 12/13 and age 19/20 CR indices into a Poisson regression. The equation yielded a pseudo-*R* ^2^ of .11 (χ^2^ = 20.85, *df* = 3, *p* < .0005). In this equation, age 12/13 CR, and no other predictor, was statistically significant. In a second step, we added age 5/6 CR to the aforementioned equation, deriving a pseudo-*R* ^2^ of .14 (χ^2^ = 24.58, *df* = 4, *p* < .0005). Age 5/6 CR, *and no other predictor*, proved significant. The 3% increase in pseudo-*R* ^2^ values between equations 1 and 2 also proved significant (χ^2^ = 6.00, *df* = 1, *p* < .025). These equations show independent prediction from the age 5/6 risk index over subsequent predictions, despite high stability of the CR index.

## Discussion

Research consistently demonstrates that CR is related to adverse outcome. This relation holds across varied domains, although CR has never been shown to influence multiple domains within a single sample studied prospectively from childhood to adulthood. We were struck by the similarity of risk items that successfully predicted this diversity of outcomes. We constructed a single CR index, hypothesizing that CR in childhood (age 5/6 years), early adolescence (age 12/13) and late adolescence (age 19/20) predicts depression, FSIQ, school dropout, smoking, arrest, and physical illness in adulthood (age 25/26). We also assessed the association between CR and CO, hypothesizing that more risk factors eventuate in more adverse outcomes. Further, we assessed whether early adversity is related to outcome independent of later adversity. Finally, we assessed whether environmental adversity interacts with S/L impairment, a powerful predictive factor intrinsic to the individual, to predict outcome, and whether CR explains unique variance beyond that explained by S/L impairment.

With complete consistency, cumulative environmental risk assessed at ages 5/6, 12/13, and 19/20, was related to all outcomes—depression, FSIQ, tobacco use, incomplete high school, arrest, and physical disease in adulthood. S/L impairment emerged as a main effect only in the context of FSIQ. This is expectable, given the verbal nature of the WAIS-III[52]. In no instance did S/L impairment interact with CR to predict an outcome. What is most striking about the findings overall is the predominant role of the CR index in predicting outcomes. Individuals with and without S/L impairments reacted similarly under conditions of CR. Therefore, we focus the remaining discussion on the association between CR and outcome.

A major finding in this study was that the greater the number of environmental risk factors experienced at all ages, the greater the number of adverse outcomes in adulthood. Indeed, CR-CO relations appeared the most reliable of all predictions. While it is difficult to compare effect sizes across regression equations, given differences in multiple, binomial, and Poisson computations, the relation between CR and CO was the only one that was consistently significant at the highest level reported here (*p* < .005), despite smaller sample size and disproportionate tendency for higher-risk participants to provide incomplete information. This is consistent with earlier literature showing that CR more powerfully predicts single outcomes than do single predictor variables [4]. It may be that CR coupled with CO provides particularly robust associations.

It is also important to note that adversity is chronic, with CR stability coefficients of .55 (Age 5/6 CR with Age 12/13 CR), .41 (Age 5/6 CR with Age 19/20 CR), and .61 (Age 12/13 CR with Age 19/20 CR; *p* < .001 in every case). These figures are comparable to that offered by Sameroff et al. [6] linking CR between ages 4 and 13, *r* = .72. These data may partly explain the difficulty involved in altering outcomes as environmental factors (and potentially underlying genetic factors related to these environmental factors) may maintain life course.

Nevertheless, early adversity accounts for variance in outcome even after later risk effects have been partialled out. We regressed age 5/6 CR on age 25/26 CO, controlling for age 12/13 and age 19/20 CR. Age 5/6 adversity scores accounted for variance beyond that explained by age 12/13 and age 19/20 scores. The amount of additional variance is small (3%). However, the numerical magnitude of an effect is not necessarily commensurate with its practical and theoretical import [73–76]. A case in point involves aspirin, recommended to reduce the probability of cardiac arrest but with an effect explaining only 3% of the variance [77]. The small numerical association between aspirin and reduced risk of heart attack is important because it applies to a large percentage of the population, across a large proportion of the lifespan, with important consequences. The same considerations are relevant to relations between childhood risk and adult outcome. Moreover, it should be noted that age 5/6 CR explained variance beyond that explained by ages 12/13 and 19/20 *despite* the fact that CR was extremely stable; this fact renders the added variance even more remarkable. In addition, age 12/13 and 19/20 CR did not explain added variance beyond that explained by age 5/6. From a theoretical perspective, there has long been debate about if and how early life experience is associated with adult outcome [78,79]. These data bear on that discussion.

There is a pressing need for theory in the CR literature [4]. The major questions posed by the present findings pertain to how a single index can predict so many outcomes, how it can predict such diverse outcomes, and why early adversity accounts for variance beyond that explained by later adversity. We cannot elucidate fully on potential mechanisms without going beyond the data, but we briefly outline a “mediated net of adversity” model that goes some way to parsimoniously integrating the findings presented in the present study.

To explain pathways from childhood to adult life, Rutter [80] introduced “chain of risk,” the notion that “the impact of some factor in childhood … sets in motion a chain reaction in which one ‘bad’ thing leads to another” (p. 27; see also [21]). Investigators also note that disadvantages cluster in time [5,9,11,81,82], consistent with the current data (Tables 1 and 2). Linking the “chain” and “cluster” observations, the present data are consistent with the notion that multiple “chains” form a web or net of adversity, with each disadvantage serving as a node to potentiate others.

However, the net of adversity model is only a heuristic or conceptual metaphor. Furthermore, it involves the implicit assumption that causal mechanisms are environmental. Moreover, as it stands, this explanation offers no inherent explanation of an important finding of the present study, that early cumulative adversity affects later outcomes, even controlling for adversity in the intervening years. We therefore suggest that factors at the nexus of the net might serve as cohesive mediating feature linking risk factors within time (explaining why risk factors cluster) and across time. As Deater-Deckard et al. [3] suggested, “diverse pathways may ultimately prove to be linked by a common core etiology” (p. 490). Of course, many mediators could potentially play such a role. But in the interests of parsimony and consistent with the current data, we are looking for a limited set of mediators that link (1) individual risk factors to one another, (2) individual outcome factors to one another, and (3) the cumulated risk and cumulated outcome indices to one another. Moreover, (4) this mediator must apply across diverse outcomes, including mood, intellect, life decisions (school dropout, smoking, arrest) and physical health. In addition, the mediators must (5) explain why adversity experienced early in life accounts for variance beyond adversity experienced later in life. These five criteria, posed simultaneously, are formidably stringent.

The mediators in question could involve neurobiological, genetic (and epigenetic), and psychological factors, as well as their interactions. By way of example only, a candidate neurobiological mechanism is allostatic load, the physiological cost of chronic exposure to stressors resulting in “wear-and-tear” on the stress system [83] (and the stability of CR shown in this and other [6] studies indicates that chronicity is relevant here). In the context of CR, we propose that each risk factor provokes added stress [1,4]. In fact, merging the CR and allostatic load constructs, Evans [11,12] showed that allostatic load increases with levels of CR exposure. Importantly, allostatic load is related to varied outcomes, including psychiatric disorder, cognitive impairment, and physical disease [11,83]. Moreover, consistent with current findings that early CR explains variance in adult outcomes beyond that explained by later CR, physiological stress responses are programmed early in life with enduring effects [79,84].

Without going into detail, other mediating processes in the CR, CO context may involve pleiotropic genes like dopamine receptor D4 (DRD4), involved in varied neurobiological processes and psychiatric/neurological phenotypes, including reaction to stress [85]. Psychosocial variables like attachment security may also serve as mediators. Attachment is linked to “an ever-widening variety of … outcomes” incorporating almost all domains of early development and beyond [86]. Moreover, meta-analytic evidence [87,88] shows that early quality of attachment continues to influence behavior into at least late adolescence, despite intervening environmental change. Speculation regarding mediated nets of adversity goes beyond the data we present. At the same time, however, the model does integrate the diverse findings reported here, meets the stringent criteria posed by these data, and provides a much-needed and flexible model for further testing.

### Study Limitations

This study has several limitations. The results pertain to a very low-risk, largely White sample [19]. This limits generalizability of findings and may attenuate relations. Replication in more broadly representative and higher-risk samples would be informative.

Furthermore, Evans et al.[4] recommended the use of multiple domains in constructing the risk index, including personal factors, immediate settings (e.g., daycare, school, neighbourhood), and cultural/societal context. In this paper, we did not include immediate settings outside the home nor did we consider broader societal issues. The addition of such features may well have strengthened our predictions further.

Another limitation involves the fact that the CR-CO analyses were missing data because Poisson regression is not amenable to multiple imputation. However, we did conduct all other regressions twice, using the imputed data in the first instance and the subsample of participants with no missing data in the second. The results were substantively comparable, suggesting that the loss of participants did not result in essential change to the findings. Again, however, replication would be useful.

Despite these shortcomings, the data do illustrate the power of CR from early childhood to early adulthood across variegated, cumulating outcomes. Equally important, the data present an explanatory challenge. We suggested that a mediated net of adversity model might meet this challenge. This model is extrapolated from, consistent with, and restricted by the data presented here; in addition, it is augmented by reliable findings from other studies. Nevertheless, the mechanics of CR remain enigmatic. The model presented briefly here, and others, require further research attention. The advantage of such efforts includes an understanding of the developmental mechanics involved in complex and synchronous development across seemingly diverse domains.
